# Production and identification of haploid dwarf male sterile wheat plants induced by corn inducer

**DOI:** 10.1186/1999-3110-55-26

**Published:** 2014-02-20

**Authors:** Wei Zhang, Ke Wang, Zhi-Shan Lin, Li-Pu Du, Hai-Li Ma, Le-Le Xiao, Xing-Guo Ye

**Affiliations:** National Key Facility for Crop Gene Resources and Genetic Improvement, Key Laboratory of Biology and Genetic Improvement of Triticeae Crops of Ministry of Agriculture, Institute of Crop Sciences, Chinese Academy of Agricultural Sciences, Beijing, 100081 China

**Keywords:** Corn inducer, Dwarf male sterile wheat, Intergeneric cross, *Ms2* haploid plant

## Abstract

**Background:**

Using the cross of wheat and maize is a very useful way to produce wheat haploid plants by chromosome elimination. Dwarf male sterile wheat (DMSW) and corn inducer are potential important germplasm for wheat breeding by recurrent selection and doubled haploid strategies. There is no report yet to achieve the haploid plants from DMSW induced by maize inbred line and especially the corn inducer.

**Results:**

Haploid plants of DMSW were successfully obtained in this study induced by both maize pollens of inducer line and normal inbred line. The efficiencies for wheat embryos formation and plantlets production induced by the two corn lines had no significant difference. All the eleven haploid wheat plants derived from the male sterile material were identified by botanic appearance, cytology, cytogenetics, and molecular markers. They were all haploid based on their guard cell length of 42.78–42.90 μm compared with the diploid control of 71.52 μm, and their chromosome number of 21 compared with the diploid control of 42. In addition, according to anthers, plant height, and molecular markers, the haploid plants were divided into two types. Eight of them showed dwarf, having no anthers, and the special band of *Rht10*, and the other three plants displayed normal plant height, having anthers, and not containing the special band of *Rht10*, indicating that they were originated from the *MS2*/*Rht10* and *ms2*/*rht10* female gametes, respectively.

**Conclusions:**

*MS2*/*Rht10* haploid plants were successfully obtained in this study by using corn inducer and inbred line, and will be employed as candidate materials for the potential cloning of *MS2* dominant male gene.

**Electronic supplementary material:**

The online version of this article (doi:10.1186/1999-3110-55-26) contains supplementary material, which is available to authorized users.

## Background

Wheat is an economic valuable crop species worldwide, and its continual genetic improvement on yield, disease resistance, processing quality, and abiotic stress tolerance is closely associated with food security production for human being (Shewry 2009; Tester and Langridge 2010; He et al. 2011). In wheat breeding program germplasm takes an essential role for the development of new varieties. Wheat germplasm with various distinguished characteristics can be identified in natural population or bred by commercial and wild cross and mutation (He et al. 2011; von Well and Ncala 2013). Of them, male sterile materials are beneficial to hybrid applications and recurrent selection of wheat (Zhai and Liu 2009). Up to date, three key male sterile genes have been found in wheat. A recessive male sterile gene, *ms1*, was found to be located on chromosome 4A (Driscooll 1978), but this gene was not convenient to be employed in wheat breeding procedure. A dominant male sterile gene, *MS3*, was obtained from the nuclear-cytoplasm hybrid between wheat and *Aegilops squarrosa* after treatment by ethyl methane sulfonate (EMS) (Maan et al. 1987; Qi and Gill 2001).

An important wheat male sterile mutant was found in 1972 by Gao (1987). Genetic analysis demonstrated that its male sterility was controlled by a single dominant gene *Tal* (Deng and Gao 1980, 1982), which was renamed as *Ms2* later (Cao et al. 2009; Zhai and Liu 2009). *Ms2* exists in wheat plants always in heterozygous status of *Ms2ms2*, and inherits itself by accepting the pollens of normal wheat varieties with *ms2ms2* genotype at this locus (Deng and Gao 1980, 1982, 1987; Gao 1987). *Ms2ms2* plants have no anthers completely while their pistils are developed normally, and their next generation always produced sterile plants by half with genotype *Ms2ms2* and fertile plants by another half with genotype *ms2ms2* (Deng and Gao 1980, 1982, 1987; Zhai and Liu 2009). By using telosomic mapping and genetic analysis approaches, this gene was located on the short arm of chromosome 4D, and 31.16 cM away from the centromere (Liu and Deng 1986a, b). To identify the *Ms2* gene and distinguish male sterile plants in early stage, a dwarf male sterile wheat (DMSW) was further developed by crossing the male sterile Chinese Spring with Aibian 1 carrying the dwarfing gene *Rht10* (*Rht-D1c*) and backcrossing the dwarfing F_1_ male sterile plants with normal Chinese Spring, in which *Ms2* gene was linked closely with *Rht10* gene with a genetic distance of 0.12 cM (Liu and Yang 1991; Zhai and Liu 2009). Since then, the dwarf male sterile wheat has been efficiently applied in wheat breeding for population improvement through recurrent selection and genetic variation expansion through easily cross-making (Liu et al. 2002), and a number of new wheat varieties have been developed by using the special material (Zhai and Liu 2009). However, *Ms2* has not been successful cloned and a perfect direct molecular marker for it has not been developed yet. The main problem is that the male sterile gene always exists in heterozygous state and the DNA samples are mixed with *ms2* from male gametes in this kind of dwarf male sterile wheat plants. Therefore, it is necessary to obtain haploid or double haploid male sterile plants derived from the *Ms2* female gametes.

Cao et al. (2009) found that *WMC617* marker was closely linked to the male sterile *Ms2* gene and the dwarfing *Rht10* gene among 48 pairs of SSR primers by bulked segregation analysis, and further mapped the two genes at the distal position of chromosome 4DS. Li et al. (2012) revealed that the *Rht10* allele contained in DMSW was generated through a tandem segmental duplication (TSD) of a region more than 1 Mb carrying *Rht2* (*Rht-D1b*) gene, resulting in two copies of *Rht2*, namely that *Rht10* was completely identical to *Rht2* in sequence. Further, they analyzed the segregation population containing *Rht10* gene by using *Rht2* gene-specific primers. It is implied that *Rht10* in DMSW also can be detected by the perfect PCR-based marker pairs of DF-MR2 developed by Ellis et al. (2002).

Wild cross of wheat and maize is the ideal way to induce female gametes originating wheat haploid plants (Laurie and Bennett 1988a). This technique has now been widely used in the production of wheat haploid plants (Laurie and Reymondies 1991; Sun et al. 1992; Chen et al. 1996a, b). Sun et al. (1999) firstly achieved the *Ms2* haploid plants by crossing the male sterile wheat with normal inbred maize line, and identified the haploid by cytogenetics. Current study is trying to obtain *Ms2*/*Rht10* haploids by using the dwarf male sterile wheat and corn inducer, and to compare the induction efficiency of corn inducer and normal inbred corn line. The obtained haploids will be identified by botanical traits, cytology, and molecular marker. Results generated in this study will be useful for marker development and isolation of *Ms2* gene, and for the production of wheat haploid plants using corn inducer.

## Methods

### Plant materials

Dwarf male sterile wheat (DMSW) line DS987 was kindly provided by Prof. Binghua Liu at the Institute of Crop Sciences (ICS), Chinese Academy of Agricultural Sciences (CAAS). Chinese Spring (CS) was demanded from the National Crop Germplasm Preservation Center at the ICS, CAAS. The pollens of corn inducer CI011-1 and Zheng58 were collected from the greenhouse of National Key Facility for Crop Gene Resources and Genetic Improvement. CI011-1 was developed by Biotechnology Research Institute of CAAS using a corn inducer, HZI1, as one parent and frequently employed for the production of maize haploid plants by the scientists in our institute (ICS, CAAS). Zheng58 was developed by Henan Xingyang Feilong Seed Limited Company of China and normally used for the induction of wheat haploid plants at ICS, CAAS.

### Crossing and hybrid embryos rescue of DMSW and corn inducer

The seeds of DMSW line DS987 were planted in the greenhouse with a photoperiod of 14 h light/10 h darkness and a temperature regime of 25°C day/15°C night. Once the heads of the male sterile plants were just completed out of the sheaths of the flag leaves, the spikes were prepared by removing the up and down spikelets and the middle florets of each remaining spikelet, and cutting the top part of the lemmas of each floret with scissors. Then, the prepared spikes were bagged with sulfuric acid paper. Three days later, the bagged spikes were pollinated with the pollens of the two maize lines by pouring from the top of the bags with hand shaking. On the next 2 days, the pollinated spikes were treated with 2,4-dichlorophenoxyacetic acid (2,4-D) 100 mg L^-1^ containing solution for 2 times (Chen et al. 1996a; Chen et al. 1996b). 14–15 days post pollination, the immature grains were collected and sterilized with 70% ethanol for 1 min, immersed in 20% bleach for 15 min and rinsed with sterile distilled water for five times inside laminar flow bench. The immature embryos were isolated with inoculating needle under anatomical lens and cultured in axial side up on germination medium (pH 6.0) containing 1/2 Murashige and Skoog (1962) (MS) basal salts, 2.0% sucrose, B5 vitamins, and 2.4 g L^-1^ phytagel at conditions of 25 ± 1°C, 16 h photoperiod, 400 μmol m^-2^ s^-1^ photosynthetic photon flux density and 45% relative humidity for 4 wk to obtain healthy haploid plantlets (Chen et al. 1996b, 2013). Finally, the plantlets were transplanted in soil in natural environment condition for further investigation. Test of percent hypothesis was used to evaluate the difference of corn inducer and normal inbred lines on inducing wheat haploid plants.

### Root tips preparation and chromosome observation

Cytological analyses followed the procedures described by Lin et al. (2002). Roots excised from the rescue plantlets and the DMSW control plants were treated in ice-water for about 24 h, fixed in ethyl alcohol and acetic acid mixture (3 : 1) for 24 h, and then transferred to 95% ethyl alcohol and stored at −20°C until use. The fixed materials were hydrolysed in 1 N HCl at 60°C for 14 min, and then stained with Schiff's reagent. Squashes of the relevant tissues were made in a drop of 1% acetocarmine on slides. Chromosome number was counted with microscope.

### DNA extraction and PCR amplification

Wheat leaves were collected from the plants of generated haploids between DMSW and corn inducer, DMSW DS987, and CS at booting stage, and their genomic DNA were extracted using modified cetyltrimethylammonium bromide (CTAB) method [3% (w/v) CTAB, 1.4 M NaCl, 20 mM ethylenediaminetetraacetic acid (EDTA), 0.1 M Tris HCl, pH 8.0, 2% (w/v) polyvinylpolypyrrolidone and 0.2% (v/v) 2-mercaptoethanol] (Mahanil et al. 2007). The DNA pellet was dissolved in sterile water.

A pair of PCR primers specific to *Rht2* gene (Accession number in Genbank: JF930281) of 5′-CGCGCAATTATTGGCCAGAGATAG-3′ and 5′-CCCCATGGCCATCTCGAGCTGCTA-3′ named as DF-MR2 (Ellis et al. 2002) were used to identify whether the freshly produced haploid wheat plants between DMSW and corn inducer contain *Rht2* gene or *Ms2* gene. PCR reaction was performed in a final volume of 20 μL consisting of 100 ng of genomic DNA, 10 uL of 2 × Taq MasterMix (CwBio, China), 3 μM of each primer and 7.4 μL of ddH_2_O. Amplification process consists of 94°C for 5 min, seven “touchdown” cycles of 94°C for 30 s, 65°C for 30 s, 72°C for 1 min 20 s with a 1°C drop in annealing temperature at each cycle, followed by 34 cycles of 94°C for 15 s, 58°C for 15 s, 72°C for 50 s, and one cycle of 72°C for 10 min. PCR products were separated on 2.0% agarose gel, and observed under UV light.

### Determination of the length of guard cells on wheat leaves

Leaf pieces of 2 cm in length were collected from each plant at jointing stage, and put on the center of a glass slide upward of the leaf abaxial epidermis. The samples were fixed with left forefinger and the mesophyll tissues were scraped off carefully by a scalpel with a little bit water until the thin achromatic epicuticula left. The stoma guard cells on the epidermis were observed and measured under an optical microscope incorporated with daily mirror micrometer (Wang et al. 1989; Du et al. 1996). Each plant was measured by ten guard cells for statistics, and the data was statistically analyzed by SPPS13.0 Software (SPSS Chicaco, USA) for significant difference and standard deviation.

## Results

### Obtaining of haploid dwarf male sterile wheat plants assisted with the pollen of corn inducer

**Figure 1:**
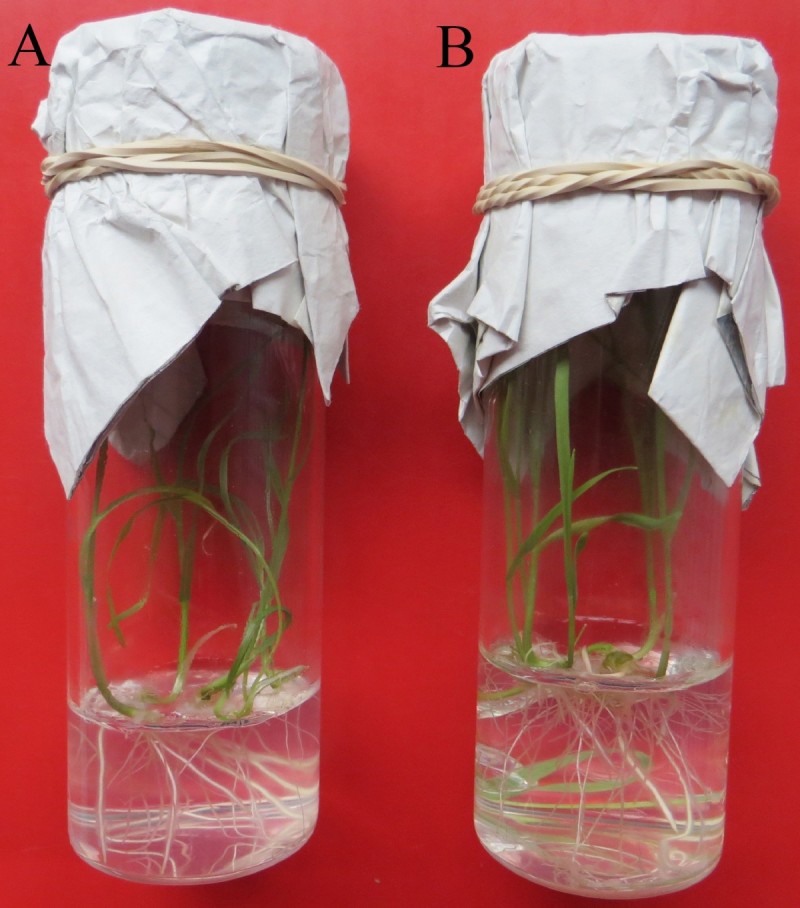
**The generated plants from the dwarf male sterile wheat induced by corn inducer and normal inbred line. A**: The plantlet was induced by corn inducer; **B**: The plantlet was induced by normal inbred corn line.

The prepared and bagged spikes of dwarf male sterile wheat line DS987 three days ago were pollinated with freshly collected pollens of corn inducer and normal inbred line. After pollination and 2,4-D treatment for 15 days, in total twenty-eight and eleven developing immature embryos were obtained from the induction of corn inducer and normal inbred line, respectively, and inoculated on the rescue medium for further growing. Finally, eight healthy plantlets were initiated by the corn inducer, and three by the normal inbred line (Figure 1). Results suggested that the frequencies for the embryos formation and plantlets induction induced by the corn inducer line (5.00% and 1.43%, respectively) were slight higher than those by the normal inbred line (3.44% and 0.94%, respectively), but the induction rate difference between the two maize lines for wheat embryos and plantlets was not significant (P>0.05) according to percent hypothesis test (Table 1).

**Table 1:** **Production of haploid dwarf male sterile wheat plants induced by corn inducer and normal inbred line**

Donors of maize pollen	Florets pollinated	Embryos cultured	Plantlets obtained	Embryos formation rate* (%)	Plantlets induction rate* (%)
CI011-1	560	28	8	5.00	1.43
Zheng58	320	11	3	3.44	0.94

*The embryo formation rate was calculated through the number of cultured embryos divided by the number of pollinated florets. The plantlet induction rate was calculated via the number of obtained plants divided by the number of pollinated florets. The rates difference between the two maize lines for wheat embryos formation and plantlets induction were not significant (P>0.05) by percent hypothesis test.

### Identifying of the haploid dwarf male sterile wheat plants by cytology and botanical appearance

**Figure 2:**
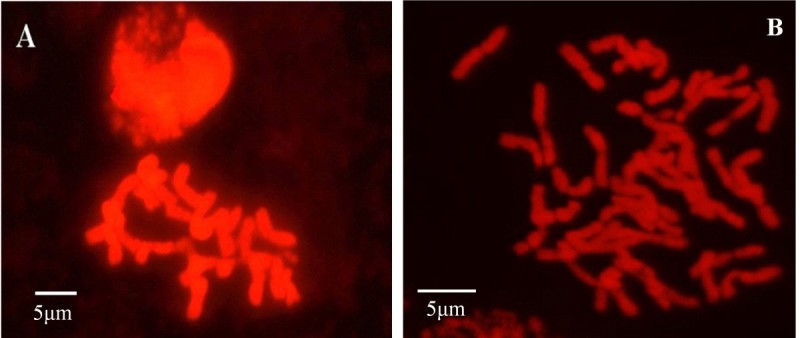
**Chromosome constitution of the rescued plants derived from the dwarf male sterile wheat induced by maize pollens and control diploid plants. A**: The rescued haploid plants contained 21 chromosomes; **B**: DS987 control plants contained 42 chromosomes.

**Figure 3:**
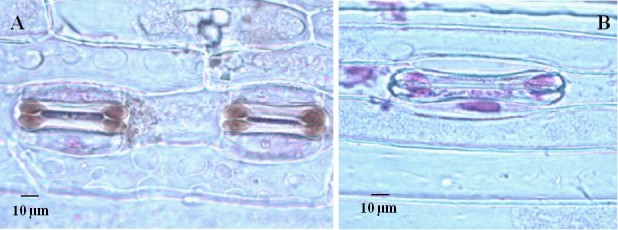
**Leaf guard cells of the rescued plants derived from the dwarf male sterile wheat induced by maize pollens and control diploid plants. A**: The rescued haploid plants had smaller guard cells; **B**: DS987 plants had larger guard cells.

**Figure 4:**
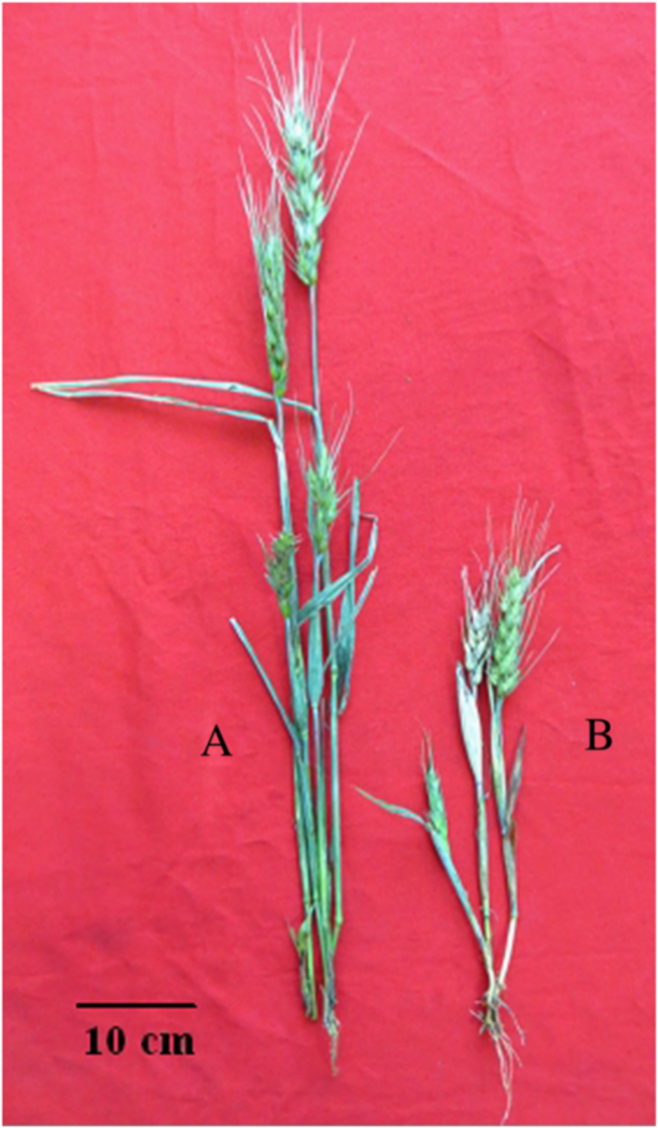
**Height appearance of the haploid plants derived from the dwarf male sterile wheat induced by maize pollens. A**: The *ms2* haploid sterile plant showed normal height; **B**: The *Ms2* haploid sterile plant showed short height.

**Figure 5:**
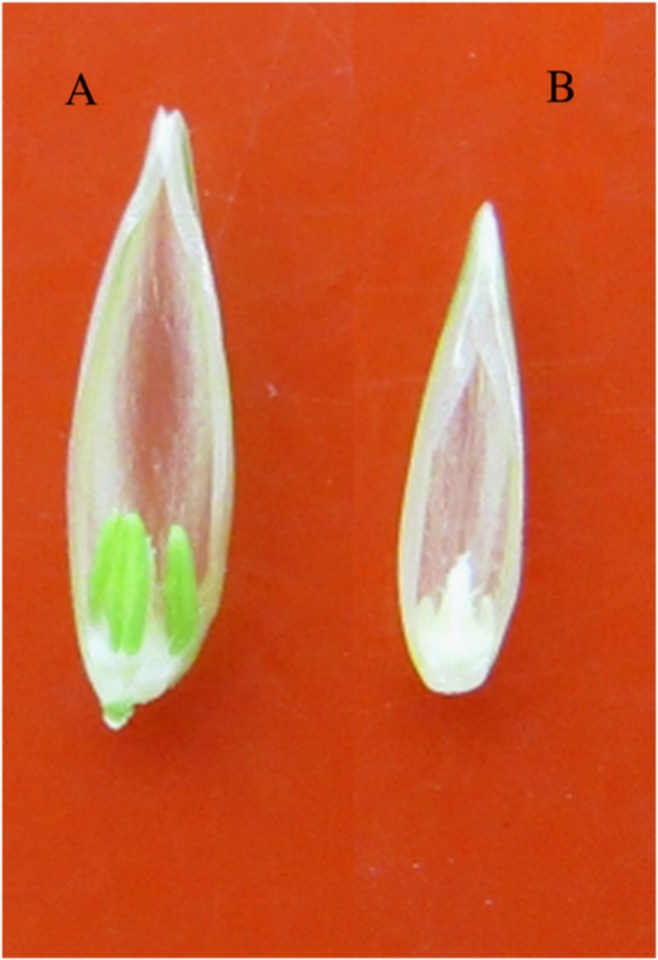
**Fertile traits of the haploid plants derived from the dwarf male sterile wheat induced by maize pollens. A**: The *ms2* haploid sterile plants had anthers but no pollens; **B**: The *Ms2* haploid sterile plants had no anthers.

The obtained eleven plants from the wild cross were examined for chromosome constitution before transplanted into soil. Twenty-one chromosomes were observed in all the rescued plants (Figure 2A), while the number was forty-two in the plants of dwarf male sterile wheat line DS987 (Figure 2B). Normally, the length of stoma guard cells on abaxial epidermis of leaves is used to primarily identify the ploidy of the plants from another culture. The size of 61.0–63.0 μm was regarded to the boundary of haploid and diploid wheat plants (Wang et al. 1989; Du et al. 1996). Thereby, the generated plants were measured for the length of guard cells on leaves at jointing stage. The average length of the guard cells was 42.90 ± 2.84 μm (varied from 40.80 μm to 46.80 μm) and 42.78 ± 1.83 μm (varied from 40.80 μm to 45.10 μm) for the short and high rescued plants, respectively (Figure 3A), which were not different significantly between the cells and plants. While, the corresponding value was 71.52 ± 4.62 μm (varied from 67.20 μm to 79.20 μm) for DS987 plants (Figure 3B). Above results demonstrated that all the generated plants from the distant cross were in haploid status. Among the eleven haploid plants, three of them were about 75 cm in plant height (Figure 4A) and had three anthers in each floret (Figure 5A), appearing normal plant height and fertile botanical traits. And the other eight haploid plants were 30 cm around in plant height (Figure 4B) and had no anthers in the florets (Figure 5B), showing dwarfing and male sterile characteristics. We believe that the haploid plants with normal height and anthers were developed from *ms2*/*rht10* female gametes, and the plants showing dwarfing and no anthers were originated from *MS2*/*Rht10* female gametes. Once induced by maize pollens, the two types of female gametes all can develop into embryos.

### Identifying of the haploid dwarf male sterile wheat plants by molecular marker

**Figure 6:**
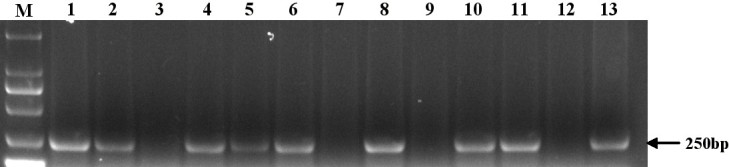
**Detection of the rescued plants derived from the dwarf male sterile wheat induced by maize pollens and control diploid plants by molecular market of DF-MR2 for** ***Rht2*** **gene or** ***Ms2*** **gene.** M: Marker ladder; 1–11: The rescued haploid plants from DMSW. The plants numbered with 1, 2, 4, 5, 6, 8, 10 and 11 showed the *Rht2*-specific band, meaning they were the haploid type of *Ms2*. The plants numbered with 3, 7, and 9 didn’t have the *Rht2*-specific band, meaning they were the haploid type of *ms2*. 12: Chinese Spring, without the *Rht2*-specific band; 13: DS987, showing the *Rht2*-specific band.

Ellis et al. (2002) developed a perfect PCR-based marker pairs DF-MR2 to detect *Rht2* gene. Therefore, all the generated haploid plants obtained in this study were detected by the molecular marker for *Rht10* and *Ms2* genes. Eight of the eleven plants amplified the specific band to *Rht10* or *Ms2*, and the other three plants didn’t amplify the specific band (Figure 6). The identification results of the haploid plants by botanical traits and molecular markers were matched in with each other, further confirming that the two groups of plants were originated from the *MS2*/*Rht10* and *ms2*/*rht10* female gametes, respectively.

## Discussion

In the intergeneric cross between wheat and maize, genotypes of the both generas exert some influences on the formation of wheat haploid embryos. Using the same maize pollens to be pollinated, different wheat lines all could produce haploid embryos with various efficiencies (Inagaki and Tahir 1990; Cai et al. 2004). For example, Chen et al. (1998) reported the embryos formed rate was ranging from 18.5% to 29.1%. The efficiencies were not only related to the growth habits of wheat varieties, but also to their origins (Laurie and Bennett 1988a; Suenaga and Nakajima 1989; Inagaki and Tahir 1990) and environmental conditions (Chen et al. 2013). However, it was demonstrated that the *Kr* gene in wheat background had no effect on the crossability of wheat and maize (Laurie and Bennett 1988b). On the other hand, a wheat line also could generate haploid embryos when pollinated with different maize genotypes, and sweet corn was better than waxy corn and normal corn in the induction efficiency of wheat haploid embryos (Suenaga and Nakajima 1989; Wang 1998; Cai et al. 2004).

Coe (1959) identified a corn inducer, Stock6, that induced parthenogernesis of maize by a higher frequency. Since then, a lot of improved corn inducers have been developed using Stock6 as parent by hybrid technique, such as WS14, Krasnodar Markers, MH1, M741H, ZMS, HZI1 (Lashermes and Beckert 1988; Chalyk 1994; Shatskaya et al. 1994; Eder and Chalyk 2002; Zhang et al. 2008; Elizabeth and William 2009). Through the use of corn inducer, plenty of haploid maize seeds can be easily obtained for its breeding (Shatskaya et al. 1994; Chang and Coe 2009; Elizabeth and William 2009; Chaikam 2012). For example, the frequency of maize haploids induced by MH1 was up to 8.0% (Eder and Chalyk 2002). However, the effectiveness of corn inducer on the induction of haploid wheat plants has not been investigated yet. Haploid dwarf male sterile wheat plants were achieved for the first time in this study by using DMSW and corn inducer. Comparing with normal inbred maize line, corn inducer didn’t show any increased induction effect on the formation of haploid wheat embryos (Table 1). It is inferred that the corn inducer cannot induce haploid plants in wheat with a high rate as it does in maize. In addition, the frequencies for haploid wheat embryos formation and plantlets induction induced by either the corn inducer or the normal maize inbred line were very low in this study comparing with other studies by inbred maize lines or hybrid maize varieties (Cai et al. 2004; Chen et al. 2013), and it might be related to the used DMSW or maize lines and environmental conditions (Suenaga and Nakajima 1989; Inagaki and Tahir 1990; Wang 1998; Cai et al. 2004; Chen et al. 2013).

Sun et al. (1999) obtained homozygote of Taigu genic male sterile wheat plants by chromosome elimination induced by maize pollens, and identified the plants by chromosome constitution. They also tried to maintain the new precious material for long term through cryopreservation by vitrification because of its sterile feature. We successfully obtained haploid dwarf male sterile wheat plants by the similar strategy, and identified the plants combining by chromosome configuration (Figure 2), cytology (Figure 3), botanical traits (Figure 4, Figure 5), and molecular markers (Figure 6). It is proved that the male sterile wheat plants only carrying *Ms2* gene can be easily achieved by the intergeneric cross of wheat and maize, and there is no need to keep the germplasm by cryopreservation. Especially, the *Ms2* haploid or homozygous plants can be conveniently recognized by the short plant height controlled by *Rht10* dwarfing gene (Figure 4), and it can be used as an ideal material to further clone *Ms2* genes in avoid of the interference of *ms2* in the diploid dwarf male sterile wheat. Next, we plan to obtain doubled haploid *Ms2* wheat plants using this approach, and analyze the plants by transcriptomics strategies such as RNA-Seq and proteomics techniques such as mass spectrometry comparing with the doubled haploid *ms2* plants derived from the same DMSW material correspondingly for the screening of differentially expressed genes and proteins between the two kinds of plants. And then, the differentially expressed genes or proteins will be confirmed by searching the sequenced wheat genomics and functionally analyzed by genetic transformation for the possible isolation of candidate *Ms2* gene.

## Conclusions

Haploid dwarf male sterile male wheat plants were obtained in this study by using corn inducer and normal inbred maize line. The efficiencies for wheat haploid embryos formation and plantlets production induced by the two types of corn lines had no significant difference. The haploid plants were further identified by botanical traits, cytology, cytogenetics, and molecular marker, and will be potentially used for the cloning of *MS2* dominant male gene.

## Authors’ original submitted files for images

Below are the links to the authors’ original submitted files for images. Authors’ original file for figure 1 Authors’ original file for figure 2 Authors’ original file for figure 3 Authors’ original file for figure 4 Authors’ original file for figure 5 Authors’ original file for figure 6

## Abbreviations

2: 4-D: 2,4-dichlorophenoxyacetic acid
CTAB: Cetyltrimethylammonium bromide
CS: Chinese Spring
DMSW: Dwarf male sterile wheat
EDTA: Ethylenediaminetetraacetic acid
EMS: Ethyl methane sulfonate
MS: Murashige and Skoog basal salt mixture
TSD: Tandem segmental duplication.
