# Supplementary figures and images for: Production and identification of haploid dwarf male sterile wheat plants induced by corn inducer
Source: Bot Stud. 2014 Feb 20;55:26. doi: 10.1186/1999-3110-55-26 (PMC5430329; doi:10.1186/1999-3110-55-26)

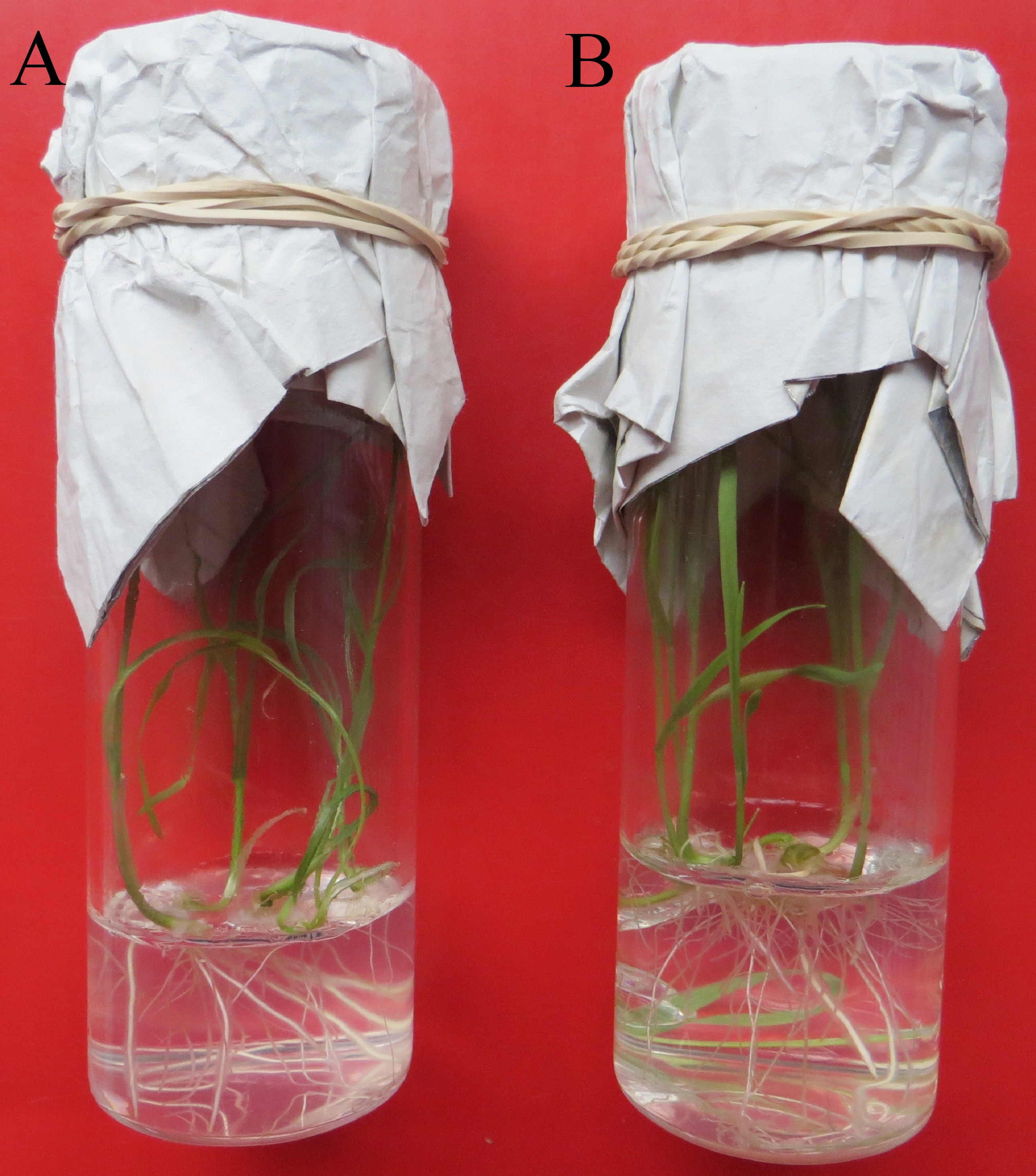

Supplement: Supplementary file 1 — Authors’ original file for figure 1 [file 40529_2013_76_MOESM1_ESM.jpeg]

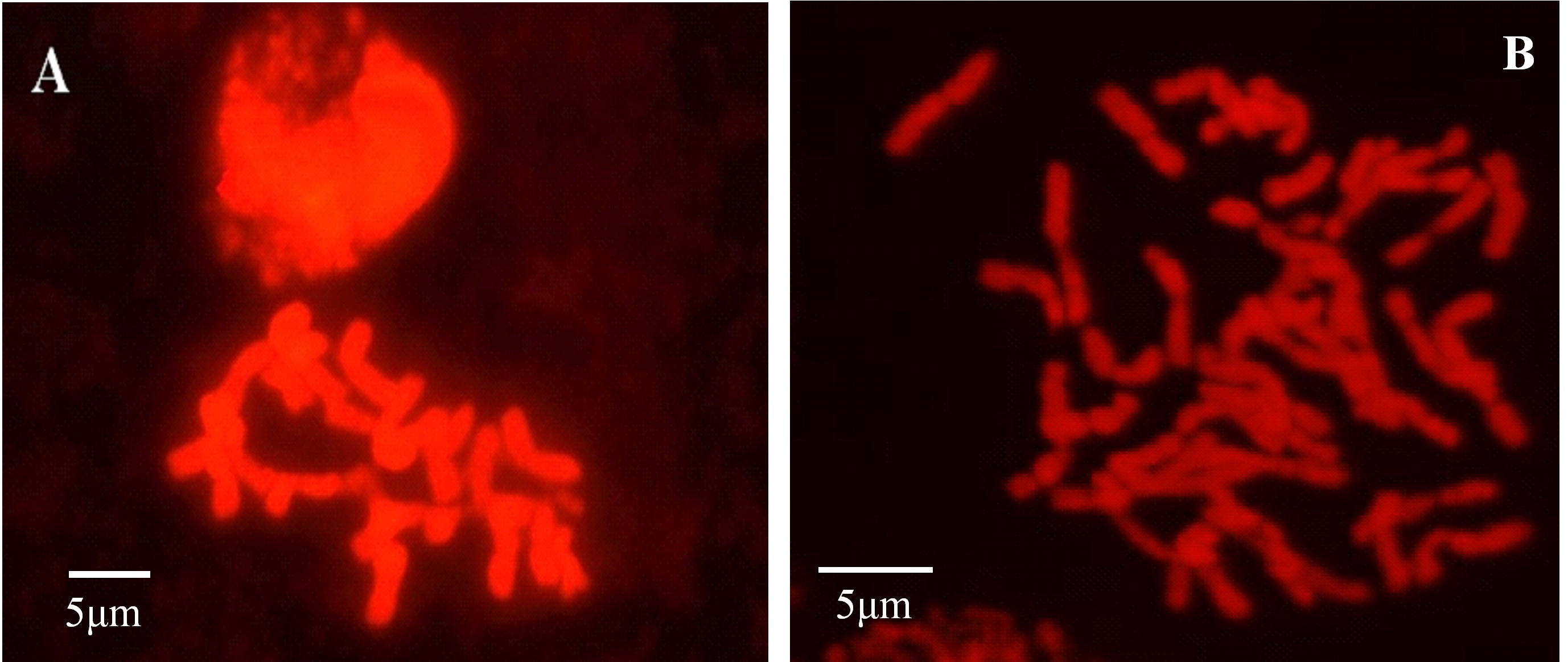

Supplement: Supplementary file 2 — Authors’ original file for figure 2 [file 40529_2013_76_MOESM2_ESM.jpeg]

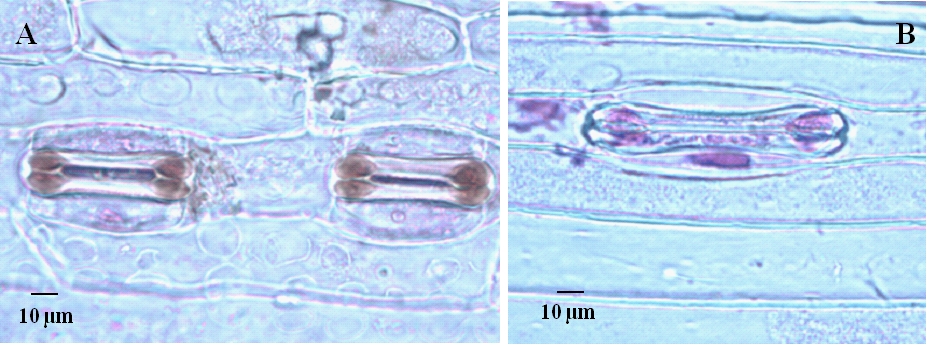

Supplement: Supplementary file 3 — Authors’ original file for figure 3 [file 40529_2013_76_MOESM3_ESM.jpeg]

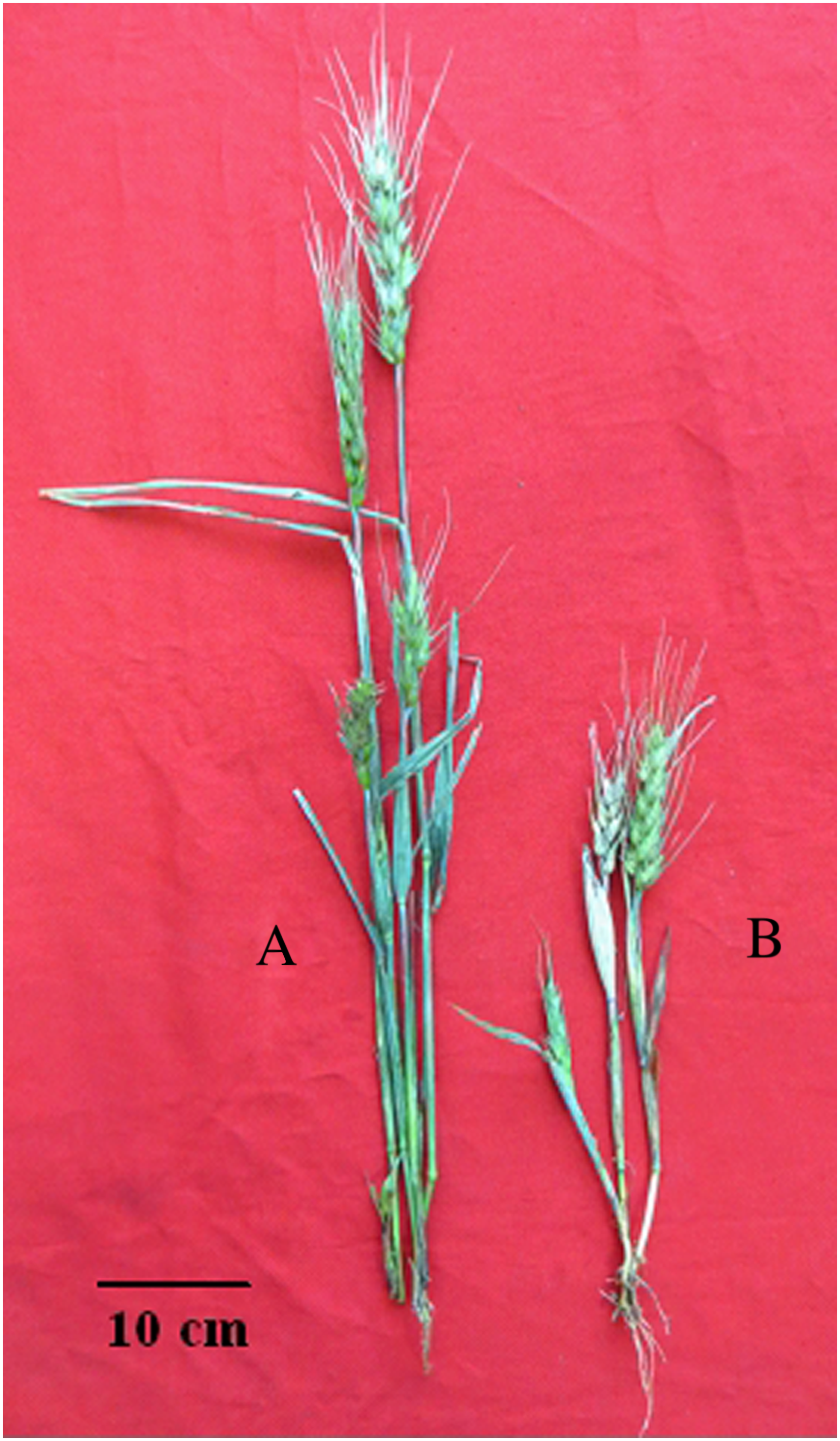

Supplement: Supplementary file 4 — Authors’ original file for figure 4 [file 40529_2013_76_MOESM4_ESM.tiff]

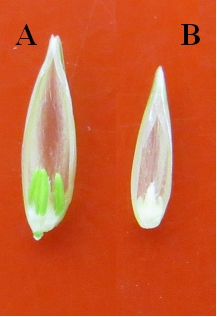

Supplement: Supplementary file 5 — Authors’ original file for figure 5 [file 40529_2013_76_MOESM5_ESM.jpeg]

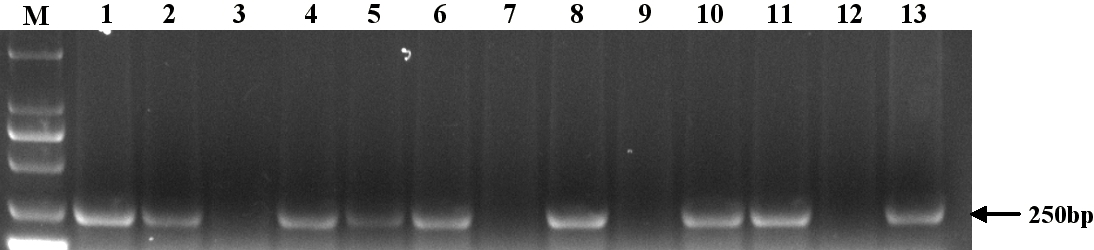

Supplement: Supplementary file 6 — Authors’ original file for figure 6 [file 40529_2013_76_MOESM6_ESM.jpeg]
